# Transplant International: Looking Back at 2025, Looking Forward to 2026

**DOI:** 10.3389/ti.2026.16267

**Published:** 2026-02-11

**Authors:** Thierry Berney, Maria Irene Bellini, Oriol Bestard, Antonio Citro, Delphine Kervella, Nina Pilat, Stefan Schneeberger, Emilien Seizilles de Mazancourt, Arianna Trizzino, Andrea Zajacova

**Affiliations:** 1 Transplant International Editor-in-Chief; 2 Transplant International Deputy Editor-in-Chief; 3 Transplant International Editorial Fellow

Transplant International (TI) has had a busy year in 2025 and the start of a new year is an opportunity to thank our authors, reviewers and editors for the quality effort consented to make TI an ever improving and impactful journal. As an indicator of TI’s growing attractivity, the impact factor has increased as have the numbers of manuscript submissions.

To our authors: Several articles published in TI in 2025, addressing various topics in the broad field of organ replacement, have been the object of considerable interest. Some papers have already been cited a few times despite the short time elapsed since publication. Congratulations to the authors who have submitted these papers and contributed to the high-quality standards of the journal (Table 1).

**TABLE 1:** Top 10 2025 most cited TI artices (Dimensions scores).

The last mile in beta-cell replacement therapy for type 1 diabetes: Time to grow up	Piemonti [1]	8 citations
Donors with previous malignancy: When is it safe to proceed with organ transplantation?	Turra et al [2]	6 citations
The evolution of immunosuppressive therapy in pig-to-nonhuman primate organ transplantation	Sanatkar et al. [3]	6 citations
Ethical issues in uncontrolled donation after circulatory determination of death: A scoping review to reveal areas of broad consensus, and those for future research	Fritz et al. [4]	6 citations
Mono-HOPE versus Dual-HOPE in liver transplantation: A propensity score-matched evaluation of early graft outcome	Koch et al [5]	6 citations
Desensitization with imlifidase for HLA-incompatible deceased donor kidney transplantation: A delphi international expert consensus	Furian et al. [6]	5 citations
Heart transplantation and donation after circulatory death in children. A review of the technological, logistical and ethical framework	Kenny et al. [7]	5 citations
Targeting CD38 in antibody-mediated rejection	Mayer et al. [8]	4 citations
Progress in porcine kidney transplantation to non-human primates	Le bas-bernardet et al. [9]	4 citations
Multi-center outcome analysis of 16 face transplantations – a retrospective OPTN study	Knoedler et al. [10]	4 citations

Measuring impact early after publication is not easy and requires other metrics than numbers of citations. Indeed, a rapid analysis on Web of Science shows that articles published in Transplant International reach their peak citation numbers the third year after publication. This is in concordance with what has been previously reported elsewhere [11, 12]. For this reason, capturing the impact early after publication requires considering other more immediate indices such as numbers of views and downloads. Expectedly, a look at the top 10 viewed or downloaded papers shows that the overlap is only partial and 9 additional articles can be identified among TI papers of highest interest (Table 2).

**TABLE 2:** Other TI articles with high views or downloads numbers (measured 8.1.26).

Liver transplantation in the context of acute-on-chronic liver failure (ACLF): Where do we stand in 2025?	L’Hermite et al [13]	8540 views 1530 downloads
Updates on donor-derived infection in solid organ transplantation, report from the 2024 GTI (infection and transplantation group) annual meeting	Eldin et al [14]	6122 views 872 downloads
The progress and challenges of implementing HLA molecular matching in clinical practice	Bezstarosti et al [15]	6100 views 684 downloads
Current techniques of gene editing in pigs for xenotransplantation	Galli [16]	5718 views 1456 downloads
State of art of dose individualization to support tacrolimus drug monitoring: What’s next?	Lloberas et al [17]	5699 views 1310 downloads
Ex-Vivo heart perfusion machines in DCD heart transplantation model: The state of art	Tessari et al [18]	5639 views 398 downloads
Nurse-led self-management support after organ transplantation – a multicenter, stepped-wedge randomized controlled trial	Van zanten et al [19]	5400 views 1579 downloads
Development of non-HLA antibodies and their association with antibody-mediated rejection in pediatric kidney transplant recipients	Schmidt et al [20]	5369 views 704 downloads
Evaluating risk in kidney living donors	Ortiz et al [21]	4797 views 1284 downloads

The topics that have generated the most interest are xenotransplantation and machine perfusion. Unsurprisingly, the same topics are found at the top of TI editors’ selection of the most impactful papers published in 2025 in the field of clinical transplantation [22]. These 2 topics have been the focus of 2 special issues that just closed at the end of 2025 and titled “Current developments in artificial organs and engineered ex-situ perfused organs” and “Europeans and Xenotransplantation” [23, 24]. The collections are now complete and will be available as e-books in the coming months.

TI is extremely grateful to the reviewers who have spent their time and expertise to assess the papers submitted to our journal. We thank them for their voluntary participation, which is key to the scientific quality of the journal. The list of the reviewers who have contributed to TI in 2025 appears at the end of this editorial (Appendix Table 3).

The biennial ESOT Congress took place in London in 2025, from June 29 to July 2, and Transplant International participated actively to the congress activities. Sessions designed and organized by the editorial board and editorial fellows discussed some of the abstracts presented during the congress as well as a selection of articles recently published in TI and covering each of the five tracks defined by the scientific program committee. A “meet the editors” workshop, aiming at the younger delegates, discussed in an interactive format several aspects of scientific publication that are not always obvious for young investigators, such as the good use of biostatistics, artificial intelligence or social media. Importantly, a collection of review and original articles based on invited lectures and best communications presented at the congress is getting ready to be completed in the first quarter of 2026 [25]. Most contributions are already published online. Highlights include reviews on immune monitoring by donor-derived cell-free DNA in heart/lung transplantation torque-tenovirus in kidney transplantation [26, 27] and on the evolution of our understanding of vascular lesions in the kidney graft biopsies [28].

The Transplant International editorial fellowship program is an ongoing success! In addition to their mentoring in all aspects of scientific editing and publishing, editorial fellows are also engaged in communication projects for the journal and have been instrumental in creating new products, such as video abstracts of TI papers and launching a TI podcast.

2025 has been a busy and productive year, but more will come the way of TI readership in 2026. From the beginning of our tenure, it has been our stated aim to strengthen the relationship with ESOT leadership and membership. We endeavor to make TI a journal close to ESOT’s style and philosophy and integrate contents resulting from ESOT’s educational and scientific activities, in the strict respect of editorial independence. Transplant International is the journal of ESOT and of the European transplant community of physicians, surgeons, scientists and allied healthcare professionals. We believe that our efforts will help increase the standing of the journal and its identification as the journal of this dynamic community!

## Appendix

**APPENDIX TABLE 3:** Reviewers for Transplant International – 2025.

**Anders Åsberg** ^a^	**Mladen knotek** ^a^	**Elisa Ruiz-Arabi** ^a^
**Olivier Aubert** ^a^	**La Salete Martins** ^a^	**Navdeep Singh** ^a^
**Alberto Benazzo** ^a^	**Johan Boble** ^a^	**Dirk Van Raemdonck** ^a^
**Klemens Budde** ^a^	**Axel Rahmel** ^a^	**Lada Zibar** ^a^
**Nicos Kessaris** ^a^	**Juliano Riella** ^a^	
Shadab Abadpour	Alexandra Geusau	René Novysedlák
Davide A. Abate	Emmanouil Giorgakis	Zhuldyz Nurmykhametova
Fariba Abbassi	Laurent Godinas	Rupert Oberhuber
Amr Abdelaal	Amanda Godoi	Jon Scott Odorico
Rayid Abdulqawi	Petra Goldsmith	Michael Oellerich
Maheen Abidi	Nicolas Golse	Grainne O’Kane
Jose M. Aguado	Jeevan Prakash Gopal	Valérie Olivier
Victoria Aguilera	Basu Gopal	Mihai Oltean
Emin Baris Akin	Julien Gras	Vivian Lumi Onusic
Mohammad Aladaileh	Paolo Antonio Grossi	Fernanda Ortiz
Varuna Aluvihare	Benoit Huery	Balin Ozsoy
Amer Alzahrani	René Hage	Alessandro Palleschi
Antonio J. Amor	Franck Halary	Eduard Palou
Dany Anglicheau	Jerome Harambat	María Paniagua-García
Corinne Antoine	Takashi Harano	Christina Papachristou
Giuseppe Aresu	Hermien Hartog	Vassilios Papalois
Miha Arnol	Koichiro Haruki	Lars Pape
Emre Arpali	Bulang He	Stefano Partelli
Florent Artru	Long He	Renato Pascale
Isa Ashoor	Ilkka Helanterä	Andrew R Pepper
Alaa Atamna	Merel Hellemons	David Pereyra
Trinidad Serrano Aullo	Rosa Blanes Hernandez	Richard Perez
Lissette Avilés	Taizo Hibi	Griffith Perkins
Alfonso Avolio	Jens Hillingsø	Marcelo Perosa
Omer Aziz	Toshihito Hirai	Quentin Perrier
Andreina Baj	Takahisa Hiramitsu	Bjoern Petersen
Chloe Balleste	Hans H Hirsch	Hessel Peters-Sengers
David a Baran	Cédric Hirzel	Palmina Petruzzo
Andrew S. Barbas	Tomer Hoffman	Paul Phelan
Louise Barbier	Benson Hoffman	Maheswaran Pitchaimuthu
Yaniss Belaroussi	Are Holm	Manuel Podesta
Caterina di Bella	Luise Holzhauser	Robert Pol
Fabrizio di Benedetto	Min Hu	Evgenia Preka
David Bennett	Volkert Huurman	Francesco Procaccio
Marius Berman	Mahmoud Mohamed Elsayed Ibrahim	Gervasio Soler Pujol
Inigo Bermejo	Franz Immer	Pankaj Puri
Dominique Bertrand	Annika Ingvarsson	Marion Rabant
Suzanne Bezstarosti	Georgina Irish	Mindaugas Rackauskas
Luigi Biancone	Michael Gerckens	Sarah Raevens
Cecilia Bonazzetti	Jasper Iske	Rajesh Rajalingam
Caroline Bonner	Fabio Ius	Sree Bhushan Raju
Antoine Bouquegneau	Manhal Izzy	Pablo Ramírez
Olivier Brugiere	Spenser January	Raja Rashid
Marcos Buj	Erik Groot Jebbink	Joanna Raszeja-Wyszomirska
Patrizia Burra	Jan Philipp Jonas	Tomas Reischig
Roberto Cacciola	Yashutosh Joshi	Jose Otto Reusing Junior
Rossana Caldara	Thomas Jouve	Jinsoo Rhu
Jasper Callemeyn	Wolfgang Jungraithmayr	James Richards
Diego Cantarovich	Ivana Juric	Paul Ritschl
Lucio Careddu	Nassim Kamar	Valentin Ritschl
Chiara Catelli	Hannah Kaminski	John Roberts
Miriam Cortés Cerisuelo	Nada Kanaan	Manuel Rodríguez-Perálvarez
Matteo Cescon	Nikolaos Karydis (Karidis)	Gianluca Rompianesi
Ernest G Chan	Martin Kauke-Navarro Md	Alberto Rosati
Xavier Charmetant	Brendan Keating	Olivier Roux
Efstratios Chatzixiros	Hussein Khambalia	Maria Jose Perez Saez
Bertrand Chauveau	Yoshitaka Kinoshita	Berta Saez-Gimenez
Yee Lee Cheah	Simon Knight	Andrew Sage
Xiaomeng Chen	Takaaki Kobayashi	Mustafa Kursat Sahin
Mikael Chetboun	Alice Koenig	Faouzi Saliba
Oriana Ciacio	Dionysios Koliogiannis	Jadranka Pavičić Šarić
Umberto Cillo	Konstantinos Koutroutsos	Marco Schiavon
Benjamin Coiffard	Philipp Dominique Kron	Roland Schmitt
Nicolina Conti	Aleksandra Kukla	Sameep Sehgal
Matthew Cooper	Vinay Kumaran	Rajeevan Selvaratnam
David Cooper	Nicholas Küng	Alp Sener
Alice Corbel	Chitaru Kurihara	Rakesh Sethapati
Emanuele Cozzi	Niclas Kvarnstrom	Sadhana Shankar
David Cucchiari	Yong Kyong Kwon	Adnan Sharif
Sílvia Gomes da Silva	Marie-Camille Lafargue	Ankit Sharma
Sunil Daga	Rita Leal	Lena Sibulesky
Raja Dandamudi	Jong Soo Lee	Farjad Siddiqui
José Davide	Per Lindner	Alberto Costa Silva
Luis Gustavo Modelli de Andrade	Sandra Lindstedt	Ksenija Vucur Simic
Marieke T de Boer	Guangxiang Liu	Rajesh Sivaprakasam
Sabina de Geest	Francesco Locatelli	Renaud Snanoudj
Margriet de Jong	Andrea Lombardi	Mario Spaggiari
Jose Luis Campo-Cañaveral de La Cruz	Ibai Los-Arcos	Peter Stock
Annelies de Weerd	Jessica Lum	Robert Stratta
Silvia Deaglio	John MacArthur	Yiliam Fundora Suárez
Rebecca Deans	Umberto Maggiore	Harikesh Subramanian
Arnaud Del Bello	Manuel Maglione	Diana Sukackiene
Tommaso di Maira	Christian T. J. Magyar	Kalathil Sureshkumar
Gonzalo Díaz-Cobacho	Beatriz Mahillo	Caner Süsal
Laura Dichiacchio	Angeles Maillo-Nieto	Andrew Sutherland
Fritz Diekmann	Rahul Mainra	Anat R Tambur
Frank J.M.F. Dor	Paolo Malvezzi	Yakup Tanriver
Didier Dorez	Nizam Mamode	Helen Te
Pedro Augusto Reck dos Santos	Wenjun Mao	Marta Tejedor
Genady Drozdinsky	Lorenzo Marconi	Inga Strand Thorsen
Geoffrey Dube	Christian Margreiter	Claire Tinel
Halise Taşkın Duman	Marco Masetti	Samuel Tingle
Richard Dumbill	Emma Massey	Francesca Tinti
Jerome Dumortier	Marie Matignon	Giusy Tiseo
Magdalena Durlik	Xavier Matillon	Pierluigi Toniutto
Philipp Dutkowski	Katharina A. Mayer	Burkhard Tönshoff
Janina Eden	Micheal Mcinnis	Lada Trajceska
Sofia el Hajji	Cely Medeiros	Vasiliki Tsarpali
Michelle Elias	Francisco lópez Medrano	Cynthia Tsien
Andreas Elmer	Zhongcheng Mei	Andreas Tzakis
Mohamed Eltemamy	Raphael Meier	David Rodriguez-Arias Vailhen
Juliet Emamaullee	Madhav C Menon	Joris Van De Klundert
Eric Epailly	Shaheed Merani	Lorena Van Den Bogaart
Olivier Epaulard	Marco Merli	Teun Van Gelder
Pauline Erpicum	Benoît Mesnard	Deborah Verran
Dilmurodjon Eshmuminov	Alberto Molina-Pérez	Ondrej Viklicky
Letizia lo Faro	Michele Molinari	Carmen Vinaixa
Mario Fernández-Ruiz	Jose Morales	Julien Vionnet
Carla Ferrandiz-Pulido	Alfonso Morcillo	Jonathan Visentin
Alberto Ferrarese	Francesc J Moreso	Fabio Vistoli
Paolo Ferrari	Midas Berend Mulder	Ashley Vo
Joana Ferrer-Fábrega	Elmi Muller	Robin Vos
Constanca Figueiredo	William Mulley	Jennifer Wainright
Konrad Fischer	Sarwa Darwish Murad	Charles-Henri Wassmer
Yohann Foucher	Silvio Nadalin	Bettina Wiegmann
Helene Francois	Nalu Navarro-Alvarez	Felicity Williams
Ilaria Gandolfini	Bashoo Naziruddin	Colin Wilson
Dale Gardiner	Flavia Neri	Tiffany Wong
Brad Gardiner	Arne Neyrinck	Germaine Wong
Cyril Garrouste	Jan Niesing	Zachary Yetmar
Philippe Gatault	Tom Nieto	Kenneth Yong
Milo Gatti	Michiel Nijhoff	Bo Yu

^a^ Special thanks to our 14 top reviewers.
